# 

**Published:** 1887-05-21

**Authors:** 


### CONTENTS.

**"And Who is my Neighbour?"** - - - 119
**Words of Consolation.**—XXXIV. The Work of the Holy Ghost- - - - - - - - 120
**Glass Eyes:** Their Manufacture and Cost. By ONE WHO HAS TO USE THEM - - - - - - - 121
**A National Pension Fund for Hospital Officials and Nurses.** The Views of Officials, Sisters, Nurses, and Others - - - - 122
**The Story of the Hospitals.**—The London Hospital (*concluded*)- - - - - - - - 123
**Chats about Medical Museums.** By ONE OF THE CROWD.—No. 5. Guy's Hospital - - - 125
**Notes and News.**—The Queen and the French Hospital.—Miscellaneous Items.—Vacancies.—Bazaar in Aid of King's College Hospital.—The Bristol Royal Infirmary.—Hospital Building Tenders.—Subscription Ball at Prince's Hall.—Friendly and Trade Societies' Demonstrations.—Donations and Legacies to Medical Charities.—Police-Sergeant Barker.—Hospital Entertainments, etc. - - - - - - - 126
**Annotations.**—The Vienna Hospital Scandal.—A Book of Grievances.—The Registration of Trained Nurses - 129
**Hospital Charity: the Best Means of Preventing its Abuse.**—By WILLIAM COLLIER, M.A., M.D., Oxford - - - - - - - 130
**A Lady Doctor on Health and Disease.**—Mrs. ANNA LONGSHORE-POTTS at Prince's Hall - - 132
**Questions and Answers.**—Matrons and Sisters at Fifty.—The Quilter Prize Form - - - - - 133
**In- and Out-Patients' Hospital Diary** - - 134
**The Children's Corner.**—Puzzles, Answers, etc. - vi
**Amusements with Profit** - - - - - vi

